# Impact of HIV-1 Infection and Antiretroviral Therapy on Bone Homeostasis and Mineral Density in Vertically Infected Patients

**DOI:** 10.1155/2019/1279318

**Published:** 2019-01-01

**Authors:** D. Donà, E. Mozzo, D. Luise, R. Lundin, A. Padoan, O. Rampon, C. Giaquinto

**Affiliations:** ^1^Division of Pediatric Infectious Diseases, Department for Woman and Child Health, University of Padua, 35100, Padua, Italy; ^2^PENTA Foundation, 35100, Padua, Italy; ^3^Infectious Diseases Department, University Hospital of Verona, 37100, Verona, Italy; ^4^Medicine Department (DIMED), University of Padua, 35100, Padua, Italy

## Abstract

Daily assumption of antiretroviral drugs and HIV-related immune activation lead to important side effects, which are particularly evident in vertically infected patients. Bone homeostasis impairment and reduction of bone mineral density (BMD) is one of the most important side effects. Primary aim of this study is to assess the prevalence of bone homeostasis alterations in a group of vertically infected patients; secondary aim is to analyze the relationship between bone homeostasis alterations and anthropometric data, severity of HIV infection, and antiretroviral therapy. We studied 67 patients with vertically transmitted HIV-1 (aged 6-31 years), followed by the Pediatric Infectious Disease Unit of the University Hospital of Padua, Italy. We analyzed bone turnover markers (P1NP and CTx) and we performed lumbar spine and femoral dual energy X-ray absorption densitometry (DXA). Personal and anthropometric data and information on HIV-infection severity and antiretroviral therapy were collected for all patients. We found that BMD values recorded by DXA showed a significant correlation with age, race, BMI, physical activity, and antiretroviral therapy duration. P1NP was increased in 43% of patients, while CTX in 61% of them. P1NP alteration was related to age, race, BMI, physical activity, therapy duration, and ever use of protease inhibitors and nucleotide reverse transcriptase inhibitors. CTX alteration was found to be correlated only with age. In conclusion, our study confirms that a wide percentage of HIV vertically infected patients show reduced BMD and impaired bone homeostasis. Strict monitoring is needed in order to early identify and treat these conditions.

## 1. Introduction

The spread of antiretroviral therapy (ART) has transformed HIV infection into a chronic condition. Daily consumption of ART and immune-activation is related to the virus itself lead to important side effects, which become particularly evident in vertically infected patients, due to life-long exposure to both drugs and virus [1]. Two important and well documented side-effects are bone homeostasis impairment and reduction of bone mineral density (BMD) [2–4].

Bone homeostasis includes the processes of bone resorption and formation and can be estimated with serum levels of bone turnover markers, which are specifically released during these processes.

P1NP (N-terminal propeptide of type 1 procollagen) is a specific marker of bone formation; it is released during type 1 collagen deposition. CTX (C-terminal telopeptide), on the contrary, is released in type 1 collagen degradation and represents a specific marker of bone resorption.

Bone mineral density (BMD) is the amount of mineral (calcium) in the bone tissue. The most used technique to assess BMD is dual-energy X-ray absorptiometry (DXA), also known as densitometry.

## 2. Aims of the Study

The primary aim of this study is to assess the presence of alterations in BMD and bone turnover (assessed by lumbar and femoral densitometry and biochemical markers) in a cohort of HIV-1 vertically infected patients.

The secondary aim is to analyze the relationship between alterations in bone remodeling and most frequent risk factors for osteoporosis (age, sex, race, BMI, smoking, and physical activity), severity of HIV infection, and ART regimen.

## 3. Materials and Methods

We conducted a single-center, cross-sectional study, from 01 January 2015 to 31 July 2015. The study was proposed to all HIV-1 vertically infected patients regularly followed by the Infectious Diseases Unit of Department for Woman and Child Health of Padua.

### 3.1. Study Population

We included all the patients followed by our Center who provided written informed consent to take part in the study.

### 3.2. Data Collection

Medical records were manually extracted and collected in an electronic worksheet specifically designed for this study.

For every patient included in the study, in addition to the regular follow-up, bone turnover markers and a bone densitometry (DXA) were collected.

Collected data include:

(i) Age, sex, and auxological parameters as weight, height and BMI.

(ii) HIV-infection severity, defined according Atlanta CDC-1994 classification [5].

(iii) Information about ART, if administered, including the duration of the single antiretroviral agent and of the combined therapy. Data about previous ART were included as well.

(iv) Smoking habits.

(v) Level of physical activity, calculated using the International Physical Activity Questionnaire (IPAQ). Metabolic Minutes (MET) have been used to assess the degree of physical activity [6].

(vi) Serum levels of procollagen type 1 N-terminal propeptide (P1NP) and C-terminal telopeptide (CTX). Reference ranges for the markers have been established according to the recommendations of the International Osteoporosis Foundation and to the International Federation of Biochemistry and Lab Medicine [7] as follows:

- P1NP: in male patients: 20-78 mcg/l; in female patients: 19-84 mcg/l;
- CTX: in male patients: > 584 ng/L; in female patients > 573 ng/L.

(vii) Bone mineral density (BMD) data, obtained through lumbar spine and femoral dual energy X-ray absorption (DXA). The classification has been carried out through the z-score value (difference between patient's BMD and the mean BMD of normal subjects with same age and gender expressed in standard deviations). Following the WHO system, we classified

(1) normal BMD: z-score < -1,

(2) osteopenia: -1< z-score < -2.5

(3) osteoporosis: z-score > -2.5

Privacy was protected in two ways: a survey number was assigned to each patient and no personally identifiable data was collected.

### 3.3. Statistical Analysis

For univariate models,* Student t* test has been used for quantitative variables and Fisher test for dichotomous variables. Multivariate data analysis was performed using logistic regression models with inclusion criterion p <0.1. For data analysis we used the software Stata v13.1, (StataCorp, Lakeway Drive, College Station, TX, USA).

## 4. Results

During the study period 67 patients were included. The population's characteristics are shown in Table 1. The mean age was 19.2 years, ranging from 6 to 31 years. Their mean exposure to ART was 13.59 years, ranging from 0 to 25 years.

At the moment of the analysis, three patients were not receiving any ART, two being naïve patients and one having suspended therapy several years before.

In 12 cases, we could not investigate previous ART history, since the patients had been followed by other centers, and no record was available.

Forty-seven (47/67, 70.1%) of the included patients underwent DXA, and 53/67 (79.1 %) completed the IPAQ questionnaire. For four P1NP value was not available.

### 4.1. BMD

Of the 67 patients, 47 underwent DXA. Their characteristics are reported in Table 2.

#### 4.1.1. Lumbar DXA

Thirty patients who underwent the test (30/47, 63.8%) presented a normal z-score; 16/47 (34%) were classified as osteopenia and 1/47 (2%) as osteoporosis. The mean z-score value is -0.18, and the variable has a normal distribution (Shapiro-Wilk test, p=0.738).

**Figure 1:**
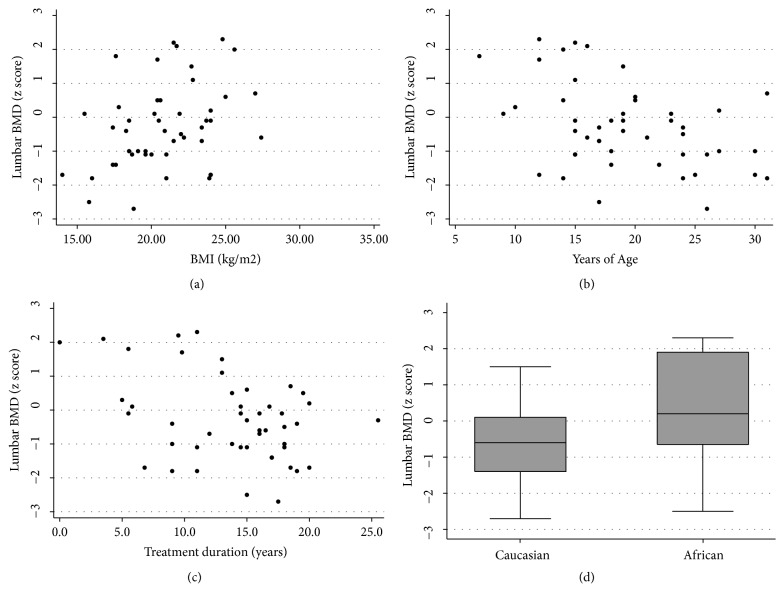
Scatter plots used to investigate the relationship between lumbar BMD z-score and BMI (a), years of age (b), and treatment duration (c). Box and whisker plot to investigate the relationship between lumbar BMD z-score and race.

In the univariate analysis, mean lumbar BMD z-scores differed significantly by race (p=0.004), while lumbar BMD z-scores were positively correlated with low BMI (p<0.001; Figure 1(a)) and negatively correlated with age (p=0.031; Figure 1(b)) and ART treatment duration (p=0.013; Figure 1(c)).

In the multivariate analysis of osteopenia or osteoporosis, higher odds of these pathologic outcomes were significantly associated with increasing age (OR 2.04; p=0.025), while lower odds of osteopenia or osteoporosis were associated with increasing BMI (OR 0.28, p=0.015) and increasing levels of physical activity (OR 0.09; p=0.044).

#### 4.1.2. Femoral DXA

Among the patients who underwent the test, 74% (35/47) presented a normal z-score, 22% (10/47) a condition of osteopenia, and 4% (2/47) of osteoporosis. The mean femoral z-score is -0.08, and the variable has a Normal distribution (Shapiro-Wilk test, p=0.197).

**Figure 2:**
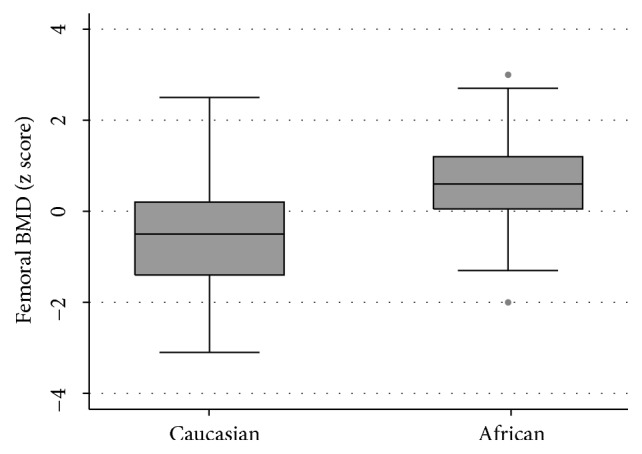
Box and whisker plot to investigate the relationship between femoral BMD z-score and race.

**Figure 3:**
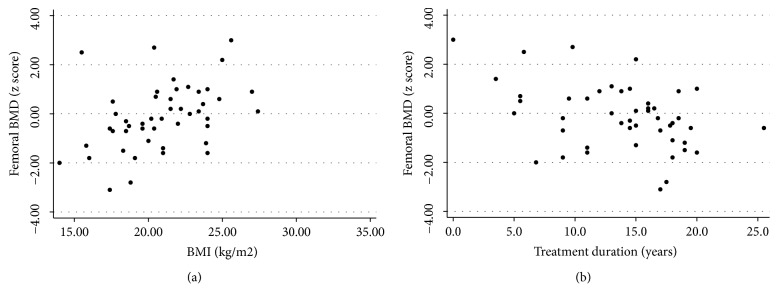
Scatter plots used to investigate the relationship between femoral BMD z-score and BMI (a) and treatment duration (b).

In the univariate analysis, similarly to the results for lumbar DXA, mean femoral BMD z-scores differed significantly by race (p=0.004; Figure 2), while femoral BMD z-scores were positively correlated with low BMI (p<0.001; Figure 3(a)) and negatively correlated with ART treatment duration (p=0.013; Figure 3(b)).

In the multivariate analysis, of osteopenia or osteoporosis, higher odds of these pathologic outcomes were significantly associated with increasing age (OR 2.04; p=0.025), while lower odds of osteopenia or osteoporosis were associated with increasing BMI (OR 0.44, p=0.004) and increasing levels of physical activity (OR 0.17; p=0.037 ).

### 4.2. Bone Turnover Markers

#### 4.2.1. P1NP

Fifty-two percent of the patients (33/63) presented a normal value; while all the remaining 48% (30/60) presented an increased value. In the univariate analysis, variables associated with an increased P1NP were age (p<0.001), African race (p=0.02), high BMI (p=0.012), long duration of ART (p<0.01), use of NRTIs (prior (p<0.001) or ongoing (p=0.03)), and use of PIs (p=0.013).

In the multivariate analysis including age, physical and clinical characteristics, and ART history and duration, the only variable that showed a significant association with P1NP is age (OR 0.65, p<0.001).

#### 4.2.2. CTX

Of the 67 patients who received the test, 39% (26/67) had a normal value, and 61% (41/67) had a value that was higher than the reference range. In the univariate analysis, age was the only variable that is significantly associated with an increased CTX value (p<0.05). No variable was significantly associated to CTX in the multivariate analysis.

## 5. Discussion

The prevalence of osteopenia and osteoporosis we reported in our cohort is similar to what has been described in other studies. Dimeglio et al, on 350 vertically infected patients (mean age 12,6 years), reported a z-score classified as osteopenia in 31% of their cases, and osteoporosis in 11% of them [8]. Similarly, another study on 98 patients with vertically transmitted HIV (age 12-20 years), in ART, described osteopenia and osteoporosis in 60% and 11% of the patients respectively [9].

De Lima et al, in their analysis on 48 patients with vertical infection (age 7-17 years), reported a mean z-score on total-body DXA of -1.19, lower than our result [10].

Other papers on pediatric patients are not comparable to ours, since they assess BMD with g/cm2, instead of using z-score.

Our analysis demonstrated a correlation between BMD and anthropometric variables, such as BMI, race, and age, if considering only lumbar z-score. It is well known that age, Caucasian race, and low BMI represent risk factors for osteoporosis [11]. Similarly female sex represents another nonmodifiable risk factor.

Our study does not show a significant correlation between z-score and sex; however we found that the only two cases of osteoporosis were female patients.

About the impact of HIV infection on BMD, we found no correlation between severe disease (CDC stage B/C) and z-score. Literature findings are inconsistent: some authors, as in our case, do not report any association between CDC stage and BMD [10], while others describe such a correlation [9, 12]. Assessing HIV severity requires both CDC stage but also CD4 and HIV-RNA count, both the nadir, and at the moment of the BMD; these parameters are used as severity indicators in several studies and show correlation with BMD. As expected, a significant correlation between ART total duration and z-score (both lumbar and femoral) was described. In our study, the mean duration of ART in the cohort who underwent DXA is 13,4 years. Even if the impact of ART in the pediatric population is not easy to interpret, considering the wide age range and the interindividual differences in pubertal development, the negative effect of ART has already been described by other authors. Dimeglio et al., in the aforementioned study conducted over a 451 pediatric patients with vertically infected HIV, 87% of whom were using ART, with a mean duration of 9.5 years, reported a correlation between ART duration and lumbar BMD (p=0.14) [8]. A similar correlation has been described by Jacobson [13].

On the contrary (to the duration of ART), no significant correlation has been found between z-score and current or past use of specific antiretroviral drugs. Several papers on pediatric patients have demonstrated the negative impact of some antiretrovirals, especially PIs, NRTIs, and tenofovir, on bone metabolism [8, 13, 14].

In our work, probably due to the low number of patients per each class of drugs, we could not highlight any relationship between BMD and the type of ART drugs.

In the multivariate analysis we found an interesting relationship between lumbar and femoral z-score and the amount of physical activity. In our population, the level of physical activity, assessed through the IPAQ is satisfactory, with more than 60% of the patients reporting a good level of physical activity, and only 9% reporting a low level. The positive impact of physical exercise in preventing osteoporosis, especially in the prepubertal and pubertal phases is well known. Physical exercise is a powerful stimulus on GH secretion. Moreover, it has been demonstrated that when sexual hormones are low (during the prepubertal phase and in the first part of the pubertal phase), physical exercise promotes the deposition of periosteal bone. On the contrary, during the last part of the pubertal phase, physical activity, combined with high level of sexual hormones, promotes the deposition of endocortical bone.

Since periosteal expansion is the major determinant of bones' resistance, it is clear that the first part of the pubertal phase represents the optimal moment to strengthen the bones' structure [15].

The protective effect of physical exercise on bone mineralization has been confirmed by other authors [8], but to our knowledge ours is the first study in which physical activity has been assessed through a validated tool (IPAQ). On the contrary, in other papers authors simply categorized physical activity in inferior or superior to the 75th percentile in their population or used a pedometer to estimate the level of activity, thus considering the walking activity only [8].

In their study, de Lima and colleagues identified the use of the pedometer for three days as such a limit to justify the lack of evidence of correlation between physical exercise and BMD [10].

In our cohort a high percentage of patients had increased value of bone turnover markers, both for P1NP (formation marker) and CTX (resorption marker).

While literature findings on adult patients provide contrasting results, showing both increase and decrease of bone formation markers [16, 17], studies on pediatric populations show an increase in both formation and resorption markers. In a study comparing 32 patients with vertically transmitted HIV, in ART (age 6.3-17.7 years), with healthy controls, Mora and colleagues reported higher values of BALP (formation marker) and NTX (resorption marker) in the HIV positive group [18]. The same authors, in another study on 40 patients with vertically transmitted HIV (35 in ART and 5 naïve), described increased values of BALP, P1NP, and NTX in the ART group, compared both to the naïve and the noninfected cohort [19]. They concluded that the increased bone turnover is the cause of the BMD reduction. About the correlation between bone turnover and ART history, even if several studies demonstrated the toxic effect of HIV and ART on the bone system, only few of them are conducted on pediatric patients with vertically transmitted infection.

The logistic regression model showed a correlation between bone markers and other variables. P1NP increase is correlated with advanced age, increased BMI, African race, overall duration of ART, past and current use of NtRTIs (tenofovir), and past use of Pis. CTX, on the contrary, has been related only to advanced age.

Studies on adult populations showed that in older ages both bone formation and bone resorption markers tend to increase: in women after menopause there is an increase both of neo-deposition markers (37-52%) and of bone resorption (79-97%). The decrease in bone mass is determined by the imbalance between bone deposition and resorption [20]. It is hence not surprising that, as reported in our study, the increasing age is followed by an increase in both P1NP (formation marker) and CTX (resorption marker).

It is known that race influences bone deposition markers, which tend to be higher in African individuals than in Caucasian ones, as reported our study, where P1NP increase is associated to African origin [21].

Similarly, the association between BMI and P1NP values is not surprising, since it is known that low BMI constitutes a risk factor for osteoporosis.

Studies analyzing the association between biochemical markers and ART are scarce, especially those conducted on vertically infected pediatric patients [19, 22, 23].

Both studies on adults and children with HIV infection show that bone markers values are usually coupled, resulting in a simultaneous increase of both bone formation and bone resorption markers. Decoupling is only observed in longitudinal studies comparing patients using ART with naive infected patients. This evidence suggests that HIV infection causes an imbalance between bone deposition and resorption, and that ART could restore bone homeostasis [24]. This could explain the correlation between tenofovir and PIs use and increase of P1NP we observed in our patients.

It is not surprising that correlation between variables and either BMD or biochemical markers is different, since DXA and turnover markers consider different aspects, representing bone metabolism, and BMD, respectively. Bone markers alterations usually precede densitometry alterations and identify even smaller modifications. As recommended by international guidelines, bone markers should be used mainly to identify a response to the treatment in patients receiving therapy for osteoporosis, providing faster and accurate information about bone status. On the other hand DXA remains the gold standard for diagnosing osteopenia and osteoporosis.

A limit of our study is the inclusion of a high number of patients in pubertal age: it is known that in this phase there is a physiological increase of bone turnover, due to the rapid statural growth. A possibility of improving this aspect could be to divide the patients in Tanner's classes before the analysis of biochemical markers. Another limit is related to the use of BMD to evaluate bone strength, which correlation with risk of fractures is unclear: we cannot therefore use low level of BMD in our HIV-patients as a predictor of pathology/fractures [25].

## 6. Conclusions

Our study confirms that a wide percentage of HIV vertically infected patients show reduced BMD and impaired bone homeostasis. Strict monitoring is needed in order to early identify and treat these conditions.

## Figures and Tables

**Table 1:** Characteristics of the study population.

Age	<10 years	4 (6%)
	10-15 years	16 (24%)
	15-20 years	16 (24%)
	>20 years	31 (46%)
	Mean (years)	19.2

Sex	Male	32 (48%)
	Female	35 (52%)

BMI	Mean (kg/m2)	21.0

Race	Caucasian	42 (63%)
	African	25 (37%)

Smoking habits	Yes	30 (45%)
	No	37 (55%)

CDC classification	N (not symptomatic)	7 (11%)
	A (mildly symptomatic)	19 (28%)
	B (moderately symptomatic)	27 (40%)
	C (severely symptomatic)	14 (21%)

Physical activity	Poor	6 (11%)
	Sufficient	11 (21%)
	Good	36 (68%)

Years of therapy	Mean (years)	13.6

**Table 2:** Characteristics of the population that underwent DXA.

Age	Mean (years)	18.8

Sex	Male	21 (45%)
	Female	26 (55%)

BMI	Mean (kg/m2)	20.7

Race	Caucasian	32 (68%)
	African	15 (32%)

Smoking habits	Yes	21 (45%)
	No	26 (55%)

CDC classification	N/A (not/mildly symptomatic)	18 (38%)
	B (moderately symptomatic)	20 (43%)
	C (severely symptomatic)	9 (19%)

Physical activity	Poor	5 (13%)
	Sufficient	9 (24%)
	Good	24 (63%)

Ongoing therapy	Fusion Inhibitors	3 (6%)

	NRTIs	43 (91%)

	NtRTIs	20 (42%)

	NNRTIs	15 (32%)

	Integrase Inhibitors	6 (13%)

	PIs	35 (74%)

Previous therapy	Fusion Inhibitors	2 (4%)

	NRTIs	46 (98%)

	NtRTIs	23 (49%)

	NNRTIs	28 (60%)

	Integrase Inhibitors	3 (6%)

	PIs	39 (83%)

Legend:

NRTIs: nucleoside reverse transcriptase inhibitors.

NtRTIs: nucleotide reverse transcriptase inhibitors.

NNRTIs: nonnucleoside reverse transcriptase inhibitors.

PIs: protease inhibitors.

## Data Availability

The data used to support the findings of this study are available from the corresponding author upon request.
